# Early Events in the Evolution of Spider Silk Genes

**DOI:** 10.1371/journal.pone.0038084

**Published:** 2012-06-22

**Authors:** James Starrett, Jessica E. Garb, Amanda Kuelbs, Ugochi O. Azubuike, Cheryl Y. Hayashi

**Affiliations:** 1 Department of Biology, University of California Riverside, Riverside, California, United States of America; 2 Department of Biological Sciences, University of Massachusetts Lowell, Lowell, Massachusetts, United States of America; University of Lausanne, Switzerland

## Abstract

Silk spinning is essential to spider ecology and has had a key role in the expansive diversification of spiders. Silk is composed primarily of proteins called spidroins, which are encoded by a multi-gene family. Spidroins have been studied extensively in the derived clade, Orbiculariae (orb-weavers), from the suborder Araneomorphae (‘true spiders’). Orbicularians produce a suite of different silks, and underlying this repertoire is a history of duplication and spidroin gene divergence. A second class of silk proteins, Egg Case Proteins (ECPs), is known only from the orbicularian species, *Lactrodectus hesperus* (Western black widow). In *L. hesperus,* ECPs bond with tubuliform spidroins to form egg case silk fibers. Because most of the phylogenetic diversity of spiders has not been sampled for their silk genes, there is limited understanding of spidroin gene family history and the prevalence of ECPs. Silk genes have not been reported from the suborder Mesothelae (segmented spiders), which diverged from all other spiders >380 million years ago, and sampling from Mygalomorphae (tarantulas, trapdoor spiders) and basal araneomorph lineages is sparse. In comparison to orbicularians, mesotheles and mygalomorphs have a simpler silk biology and thus are hypothesized to have less diversity of silk genes. Here, we present cDNAs synthesized from the silk glands of six mygalomorph species, a mesothele, and a non-orbicularian araneomorph, and uncover a surprisingly rich silk gene diversity. In particular, we find ECP homologs in the mesothele, suggesting that ECPs were present in the common ancestor of extant spiders, and originally were not specialized to complex with tubuliform spidroins. Furthermore, gene-tree/species-tree reconciliation analysis reveals that numerous spidroin gene duplications occurred after the split between Mesothelae and Opisthothelae (Mygalomorphae plus Araneomorphae). We use the spidroin gene tree to reconstruct the evolution of amino acid compositions of spidroins that perform different ecological functions.

## Introduction

**Figure 1:**
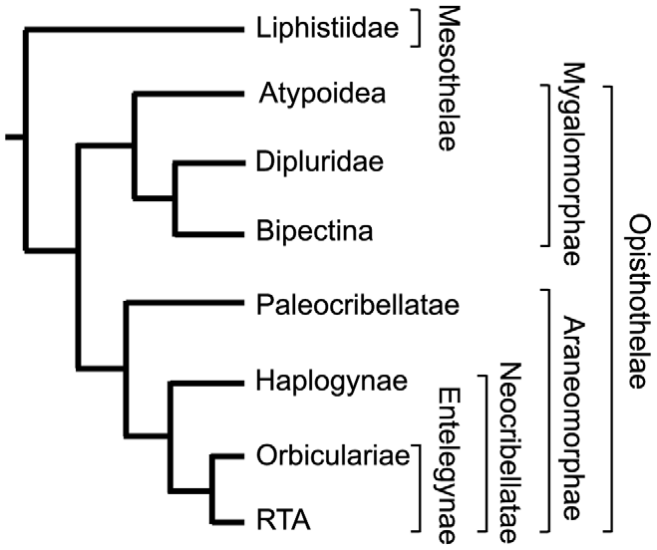
Phylogeny for spider groups analyzed in this study. Phylogeny is based on [2], [48].

**Figure 2:**
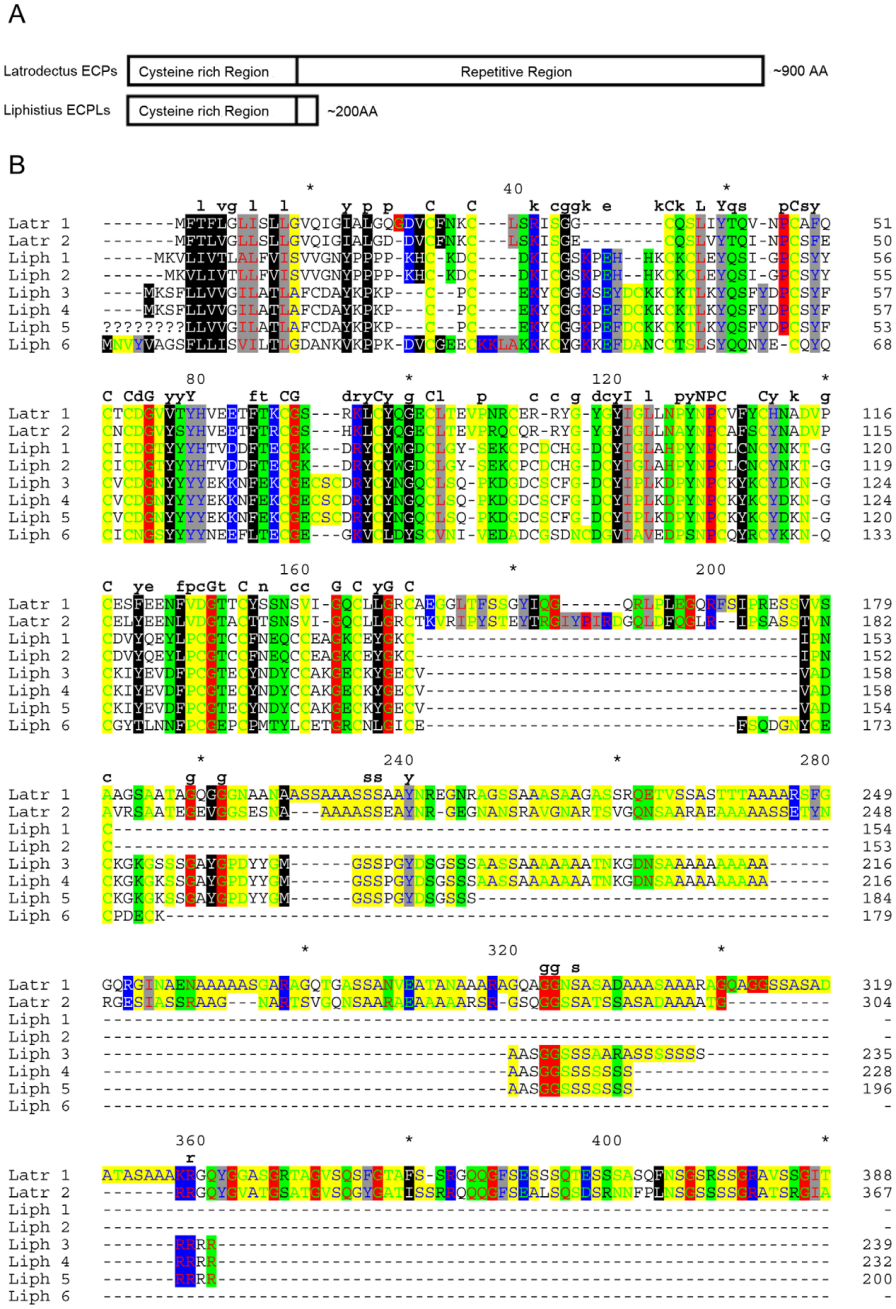
Alignment of Egg Case Proteins (ECPs) and Egg Case Protein-like proteins (ECPLs). A) Schematic of alignment of *Latrodectus hesperus* ECPs and *Liphistius malayanus* ECPLs. B) Alignment of amino acid sequences, abbreviated using single letters. Only partial *Latrodectus* (Latr) ECPs are shown as *Liphistius* (Liph) ECPLs lack the extended repetitive region. Alignment columns were highlighted using GeneDoc [67] according to physiochemical properties (Text color/Shade color: Proline Blue/Red; Glycine Green/Red; Tiny Blue/Yellow; Small Green/Yellow; Positive Red/Blue; Negative Green/Blue; Charged White/Blue; Amphoteric Red/Green; Polar Black/Green; Aliphatic Red/Gray; Aromatic Blue/Gray; Hydrophobic White/Black). Upper-case single letters occur above alignment positions showing 100% amino acid conservation, while lower case single letters occur above positions showing >50% conservation.

Silk is vital to the ecology of spiders, being used throughout their lifetime for a wide array of essential functions. There are over 42,000 described species of spiders [1], and they are not only taxonomically diverse but also ecologically diverse in their silk biology. Yet few species have been sampled for their silk genes. While most silk research has focused on derived members of Araneomorphae (“true spiders”), we present silk genes from Paleocribelletae (a basal araneomorph clade), increase sampling for Mygalomorphae (trapdoor spiders, tarantulas, and their kin; the sister group to Araneomorphae), and record silk sequences from Mesothelae (segmented spiders; the sister suborder to all other spiders; Figure 1; [2]). Mesotheles and mygalomorphs exhibit profound differences in silk use compared to most araneomorph spiders [3], [4]. Mesotheles and mygalomorphs produce general-purpose fibers and apply silk in a sheet-like manner to a burrow or other substrate, which is believed to be most similar to silk use in the common ancestor of extant spiders that lived >380 million years ago [2], [5]–[11].

Spider silk is known for its extraordinary mechanical properties, rivaling most natural and synthetic materials in strength, flexibility, and toughness [12]–[15]. Silk is chiefly composed of proteins known as spidroins (a contraction of spider fibroins; [16]), which are encoded by members of a multigene family [17]–[23]. Studies on the spidroin gene family in orbicularian spiders show that these proteins are very long (up to 15 kb) and highly repetitive [24]–[28]. The composition of the repetitive protein-coding region is often dominated by a few amino acids - particularly alanine, glycine, and serine. The amino acid composition of the repeat regions varies considerably across different spidroin gene family members, and plays an important role in the mechanical properties of the different silk fibers [29].

Egg Case Proteins (ECPs) comprise a second class of proteins found in spider silk but have been identified only in the egg cases of the Western black widow, *Latrodectus hesperus* [30], [31]. Unlike spidroins, ECPs are rich in cysteine. The cysteines are hypothesized to form disulfide bonds with tubuliform spidroins, the major component of *Latrodectus* egg cases [31]. Since ECPs are only known from a single species, the evolutionary history of this gene family is not clear. The phylogenetic distribution of ECPs suggests that the genes that encode ECPs are a recent evolutionary innovation restricted to black widow spiders.

Silk gland morphology and silk fiber use in mesothele, mygalomorph, and paleocribellate spiders are relatively simple in comparison to that of orbicularian (orb-web weaving) spiders [6], [32]–[36]. Orb-weavers produce individual silk fibers that are task-specific, such as major ampullate silk, which is used in draglines and aerial orb-web frames, and tubuliform silk, which is incorporated into egg cases. Orb-weaver spiders produce up to seven silk types with unique functions that are synthesized in different morphologically distinct glands [29]. In contrast, mesotheles and mygalomorphs generally have morphologically indistinct glands that do not produce task specific fibers. Therefore, characterizing silk transcripts in mesotheles, mygalomorphs, and a basal araneomorph lineage allows for a better understanding of the evolutionary transition from substrate-borne, general-use silk fibers to aerial webs with task specific fibers spun by orb-weavers.

Despite the simplicity of their silk gland morphology and fiber types, mesothele and mygalomorph spiders rely heavily on their silk. Silk is crucial for extending the prey detection sensory area [3]. Additionally, these spiders are long lived and may inhabit a single burrow for their entire life (10–20 years; [37]), making durable silk important for burrow maintenance. Different species of mesotheles and mygalomorphs construct a variety of web types (e.g., sheet-webs and purse-webs) and burrow entrance architectures (e.g., trip-lines, turrets, and trapdoors) indicating the potential for the discovery of silk proteins with unique mechanical properties. Further, histological studies of silk glands from representatives of basal spider lineages suggest the production of multiple protein types, raising questions regarding the silk gene diversity in these spiders [6], [32]–[35].

Spiders have received considerable attention because of the high-performance silks that they produce and the variety of ways that these silks are deployed in different ecological and behavioral contexts; yet, the understanding of the origin and early evolution of spidroins and silk remains limited [38]. Additionally, little is known about the diversity of silk encoding genes across spider phylogeny. Characterizing silk genes from Mesothelae, the sister group to all other extant spiders, is essential for this purpose. The few studies that have characterized mygalomorph silk genes indicate that spidroins diversified prior to the mygalomorph/araneomorph split, and mygalomorphs have the potential for producing multiple spidroins [21], [39], [40]. The recent controversy regarding silk production in the tarsi (terminal leg segments) of tarantulas also highlights the need for further investigation into the diversity of silk proteins in these spiders [41]–[45].

We constructed cDNA libraries from the silk glands of spiders from Mesothelae, Mygalomorphae, and Paleocribelletae, for the purpose of characterizing the genes encoding their silk proteins. We found a considerable diversity of silk associated cDNAs in the mesothele species, *Liphistius malayanus*; in particular, we discovered homologs to ECPs that are otherwise only known from the orbicularian species, *Latrodectus hesperus*. Also, we infer from a reconciliation analysis of our spidroin gene tree that gene duplications occurred in the common ancestor of opisthotheles, after they split from mesotheles. Ancestral state reconstruction of spidroin repetitive region characteristics on the spidroin gene tree was used to infer evolutionary transitions in repeat sequence that have led to specialized and functionally diverse fibers in spiders.

## Results

### *Liphistius* Egg Case Protein Homologs

BLASTX searches [46] of cDNA clones identified six *Liphistius malayanus* transcripts with top hits to *Latrodectus hesperus* ECP1 (AY994149) and ECP2 (DQ341220). Thus, these *Liphistius* transcripts were named ECP-like (ECPL; GenBank accessions JX102548-JX102553). No ECP-like transcripts were detected in any of the mygalomorph cDNA libraries or the *Hypochilus thorelli* cDNA library. *Liphistius* ECPL names and cDNA lengths in base pairs (bp) in parentheses are as follows: ECPL1 (836), ECPL2 (724), ECPL3 (967), ECPL4 (969), ECPL5 (800), ECPL6 (950). With the exception of ECPL5, all of the *Liphistius* ECPL mRNA sequences included full length coding sequence. *Liphistius* ECPL transcripts are significantly shorter than *Latrodectus* ECP1 and ECP2 transcripts, which are 2799 bp (coding, 932 amino acids (AA)) and 2478 bp (coding, 825 AA), respectively. The *Liphistius* ECPLs align to the non-repetitive, cysteine rich, N-terminal region, and lack most of the repetitive region of the *Latrodectus* ECPs (Figure 2a). The average pairwise similarity for amino acid sequences (gaps treated as missing) among *Liphistius* ECPLs is 58.26%, and 33.53% between *Liphistius* ECPLs and *Latrodectus* ECPs (Figure 2b).

### Spidroin Gene Tree

One or more spidroins were identified in the cDNA libraries for each taxon in our study, for a total of 13 new spidroins (GenBank accessions JX102554-JX102566). All of the spidroin cDNAs were partial length transcripts, lacking 5′ untranslated sequence, a start codon, N-terminal region sequence, and an unknown amount of repeat region sequence. Spidroin names with cDNA lengths (bp) in parentheses are as follows: *Liphistius* fib1 (3513); *Hypochilus* fib1 (2063) and fib2 (2190); *Aphonopelma seemanni* fib1 (1904), fib2 (1634), and fib3 (1464); *Poecilotheria regalis* fib1 (4617) and fib2 (2437); *Antrodiaetus riversi* fib1 (1833) and fib2 (5023); *Sphodros rufipes* fib1 (2460); and *Hexura picea* fib1 (409). *Megahexura fulva* fib1 (1257) contained a C-terminal encoding region but lacked a complete repeat; therefore, an additional clone (4897) of exclusively repetitive region was sequenced. Comparison of the repeat regions of these two clones confirmed that they likely represent parts of the same transcript. The two *Megahexura* fib1 clone sequences were combined in GenBank accession JX102566.

**Figure 3:**
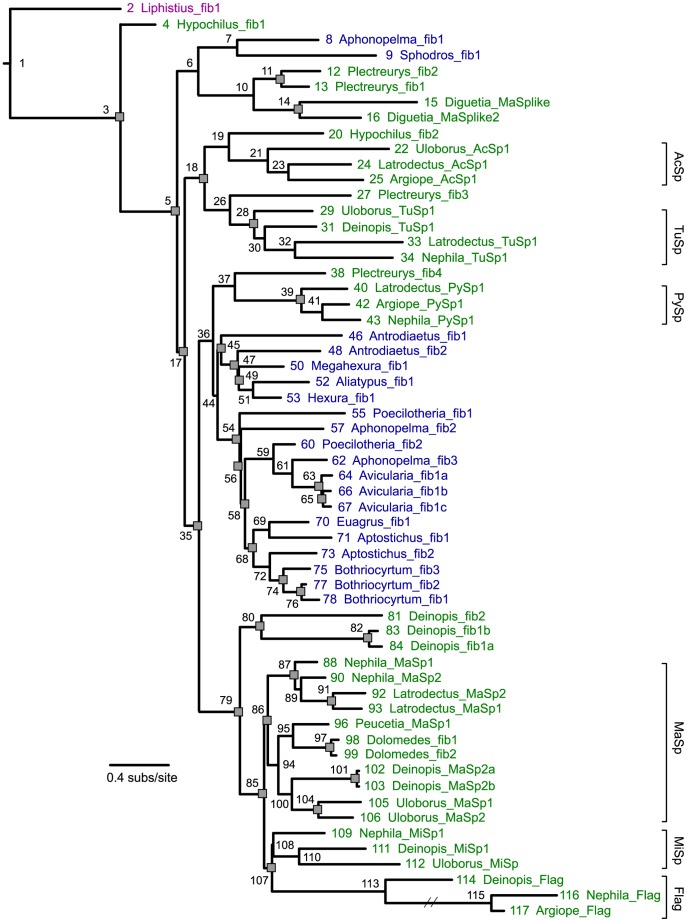
Spidroin gene tree with inferred duplication events. Spidroin gene tree is based on a ML analysis of the carboxy-terminal encoding region with gaps coded as binary characters and monophyly of some groups constrained (see Methods). Numbers next to nodes and terminals correspond to numbers in supplementary Tables S1 and S2 showing support values, alternate rootings, and continuous character data. Spidroins are colored according to the taxonomic group from which they were characterized: purple = Mesothelae, blue = Mygalomorphae, green = Araneomorphae. Gray squares indicate duplication events inferred by reconciliation. Hash marks on branch indicate arbitrary shortening of branch for figure quality purposes. Brackets indicate clades with the following abbreviations: AcSp = Aciniform, TuSp = Tubuliform, PySp = Pyriform, MaSp = Major ampullate, MiSp = Minor ampullate, Flag = Flagelliform.

We used the tree based on the maximum likelihood (ML) analysis with constraints (Figures 3, S1; Table S1) for reconciliation analysis and reconstruction of the evolution of continuous characters (Table S2). While tubuliform, aciniform, pyriform, and flagelliform spidroins were each recovered as monophyletic in all ML and Bayesian analyses, without these constraints, monophyletic groupings of neither major ampullate spidroins nor minor ampullate spidroins were recovered. However, monophyly of both major ampullates and minor ampullates is supported by a previous Bayesian analysis of combined N and C-terminal data [23]. The ML constrained and unconstrained trees were identical at 46 of 58 nodes (Figure S1). Conflicting relationships were restricted to weakly supported nodes (Table S1). The Shimodaira-Hasegawa (SH) test [47] determined that the constrained topology was not significantly worse than the unconstrained topology. Both the constrained and unconstrained Bayesian consensus trees were unresolved at many nodes (Table S1). The ML and Bayesian constrained trees conflicted at only one node, where MiSps were placed sister to Flags in the ML analysis but sister to MaSps in the Bayesian analysis. The bootstrap percentage and posterior probability were weak for either relationship.

The modest support at many nodes on the spidroin gene tree is not surprising given the small character set available (only C-terminal encoding regions) and the deep divergences among the taxa sampled. Support values for nodes of the spidroin gene tree will likely be improved in the future with inclusion of N-terminal regions, which are available for only a limited subset of published spidroins [23]. Our spidroin gene tree is generated from the broadest phylogenetic sampling of spider lineages to date and thus is the best available topology for reconciliation and ancestral character state reconstruction analyses.

Reconciliation analysis of the spidroin gene tree with the species tree supported the *Liphistius* spidroin as sister to all other spidroins (100 events = 31 duplications+69 losses; Figure 3; Table S2). Twenty-five other rootings implied the same number of duplications, but at an increased loss cost. Alternative rootings with *Hypochilus* fib1, or *Hypochilus* fib1 plus *Liphistius* fib1, resulted in the next best reconciliation score (101 events = 31 duplications+70 losses) compared to the optimal score (rooting with *Liphistius* fib1).

Reciprocally monophyletic araneomorph and mygalomorph spidroin groups were never recovered in the phylogenetic analyses. Based on the most parsimonious rooting, *Hypochilus* fib1 was found to be sister to all remaining opisthothele spidroins, while *Hypochilus* fib2 was placed sister to the orbicularian aciniform spidroins (Figure 3). Mygalomorph spidroins fell into two groups. The most basal mygalomorph group consisted of a tarantula spidroin (*Aphonopelma* fib1) and an atypoid spidroin (*Sphodros* fib1), and this clade of genes was sister to spidroins from the haplogynes, *Plectreurys* and *Diguetia*. Most mygalomorph spidroins clustered in a group that was sister to an araneomorph clade consisting of *Plectreurys* fib4 and all of the orbicularian pyriform spidroins. This second mygalomorph clade is characterized by a basal split between atypoid spidroins and non-atypoid sequences; however, relationships within these two groups did not necessarily follow accepted species relationships [48].

### Spidroin Repeats

**Figure 4:**
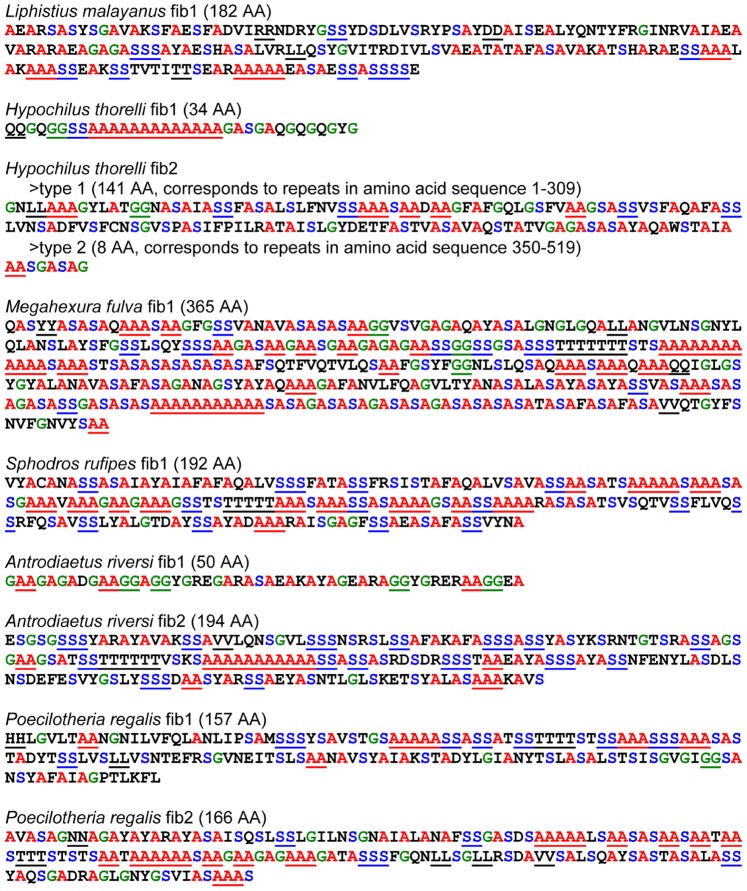
Majority rule consensus of ensemble repeats within spidroins. Ensemble repeats are tandemly arrayed. Amino acid sequences with single letter abbreviations are shown. Alanine (red), serine (blue), and glycine (green) are highlighted. Single amino acids repeated in tandem are underlined. Repeat lengths are given in parentheses.

XSTREAM [49] analyses identified repeat sequences in 9 of the 13 newly characterized spidroins (spidroin sequences *Aphonopelma* fib1, *Aphonopelma* fib2, *Aphonopelma* fib3 and *Hexura* fib1 were too short to record iterated repeats). Consensus repeats and their lengths are displayed in Figure 4. Most consensus repeat lengths are between 140 and 200 AA. *Hypochilus* fib1 and *Antrodiaetus* fib1 are significantly shorter at 34 and 50 AA, respectively. XSTREAM identified two repeat types in *Hypochilus* fib2. The consensus length of the first type, corresponding to repeats found in residues 1–309, is 141 AA. In contrast, the consensus repeat length of type two is 8 AA, and corresponds to repeats within residues 350–519. The *Megahexura* fib1 consensus repeat, at 365 AA, was significantly longer than the repeats from the other newly characterized spidroins described here. Unlike *Euagrus* fib1, which has a repeat of similar length (342 AA; [18], [21]), the *Megahexura* fib1 repeat could not be broken down into sub-repeats of approximately ∼180 AA in length.

**Figure 5:**
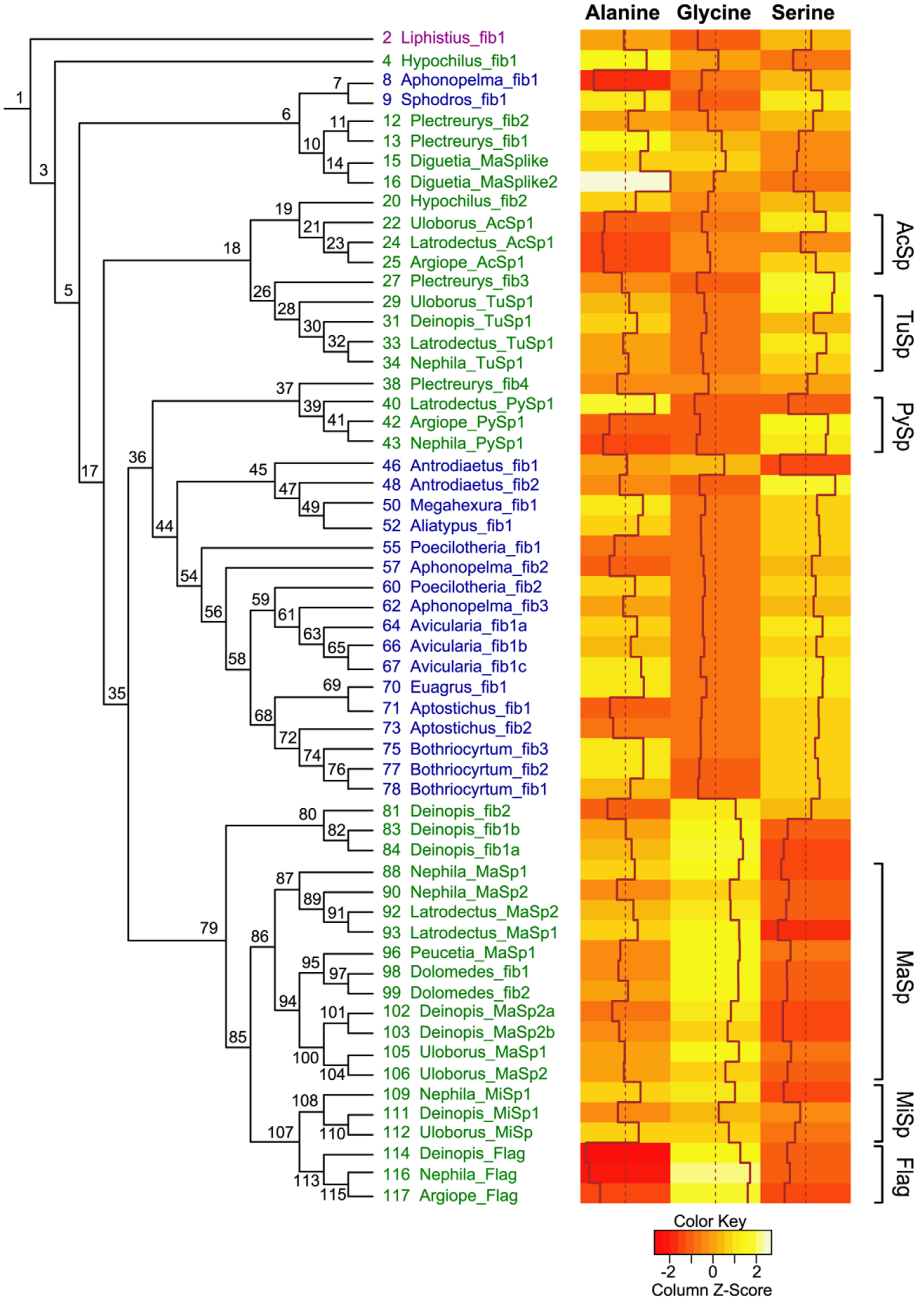
Heat map of percent compositions of alanine, glycine, and serine from spidroin repetitive regions. Cladogram adjacent to heat map shows relationships as in Figure 3. *Hexura* fib1 was omitted since no repetitive region sequence was obtained for that cDNA. Here, red indicates levels furthest below the mean, while white indicates levels furthest above the mean. Histograms on columns also show relative composition levels of the three amino acids across spidroins. Spidroin colors and abbreviations for clade names are as in Figure 3. Numbers at nodes correspond to information in supplementary Tables S1 and S2.

Repeat regions of most spidroins reported here are rich in alanine and serine, but low in glycine (Table S2). Proline, which is implicated in the extensibility of orb-weaver major ampullate and flagelliform silks [50], is rare in the spidroins reported here as well as in previously reported mygalomorph spidroins (0–4.13%). Alanine and serine tandem repeats occur in all of the newly generated spidroin sequences, whereas iterations of other amino acids are less common (Figure 4). The repeat region compositions of alanine, glycine, and serine for all spidroins analyzed in this study are summarized in Table S2. The individual contributions of alanine, glycine, and serine relative to the total composition for each spidroin are displayed in a heat map (Figure 5). Alanine levels are variable across spidroins. Glycine and serine levels appear to trade-off with each other in that they exhibit large and opposite changes. Glycine deficiency and high serine levels are primarily found in *Liphistius*, mygalomorph, and haplogyne spidroins, as well as tubuliform, aciniform, and pyriform gland-associated spidroins. By contrast, *Deinopis* fib1a and fib1b along with major ampullate, minor ampullate, and flagelliform gland-associated spidroins, have high glycine levels but are deficient in serine.

Continuous character modeling of alanine, glycine, and serine amino acid compositions, given our preferred tree (Figure 3), were executed using CoMET [51]. Optimal models (pure phylogenetic, non-phylogenetic, or punctuated average, in combination with distance, equal, or free; [52]) were chosen by the Akaike Information Criterion [53]. For alanine composition, the punctuated average/equal model was selected under the asymmetry threshold of 100, but the pure-phylogenetic/distance model was selected under the asymmetry threshold of 1000. The punctuated average/equal model was selected for glycine composition under thresholds of 100 and 1000. The model selected for serine composition was pure-phylogenetic/distance under both thresholds.

**Figure 6:**
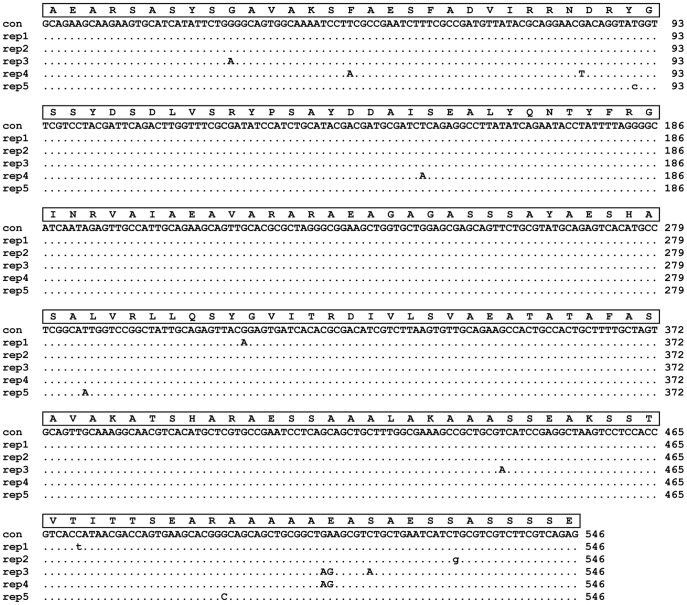
Alignment of DNA sequences for *Liphistius* fib1 repeats. Amino acid translation and DNA consensus sequences are above repeat sequences. Dots indicate identity to the consensus sequence. Non-synonymous and synonymous differences from the consensus are indicated by upper and lower case letters, respectively.

For each of the newly characterized spidroins, comparison of DNA sequences across repeats of a particular molecule reveal a high degree of sequence similarity among repeats. *Hypochilus* fib1, fib2 repeat 1, and fib2 repeat 2 showed the lowest average percent identities across repeat types at 85%, 79% and 77%, respectively. Repeats in the mygalomorph spidroin, *Antrodiaetus* fib1, shared 87% identity. Repeats within each of the six other new spidroins with identifiable repeats were >98% identical. A very low total of 13 non-synonymous differences and 3 synonymous differences occur across the 546 bp long alignment of *Liphistius* fib1 repeats (Figure 6).

## Discussion

### *Liphistius* Silk Gene Diversity

The common ancestor of mesotheles and all other spiders is estimated to have existed more than 380 million years ago [10]. This deep divergence and distant phylogenetic relationship with other spiders makes characterization of silk genes from Mesothelae crucial for obtaining a complete understanding of silk evolution. Mesotheles retain a number of plesiomorphic morphological characters associated with silk spinning (e.g., four pairs of anteriorally-placed spinnerets and single spigot types), and these spiders exhibit little variation in silk fiber types [4], [6]. However, mesotheles use silk for a variety of functions such as construction of their egg cases, burrow, trapdoor, and sensory lines [6], [38]. This combination of silk-spinning traits raises questions about the underlying diversity and function of silk genes and proteins from Mesothelae.

The *Liphistius* cDNA library included a considerable diversity of silk protein transcripts. In total, seven silk associated cDNAs were recovered, which approaches the number of different ortholog groups described from a single orb-weaver species and surpasses the number reported from most non-orbicularian araneomorph species [18], [22], [23]. This diversity is surprising given the much simpler silk gland morphologies of *Liphistius* compared to araneomorph spiders. Six of the seven *Liphistius* silk cDNAs shared substantial sequence similarity to the ECPs (egg case proteins; BLASTX E values <1e-05), which have thus far only been reported from the Western black widow, *Latrodectus hesperus* [30], [31]. The six *Liphistius* egg case protein-like (ECPL) sequences group into three clusters. DNA sequence percent similarities across these three groups range from 49–57%. Within groups, percent similarities (gaps treated as missing) range from 96–100%. All of these sequences exhibit length differences in the protein-coding region, and for one of the groups, the only difference between members was a three-base pair indel. It is possible that some of the ECPL sequences represent allelic differences and/or splice variants.

The phylogenetic distribution of ECPs and ECPLs implies that egg case proteins either convergently evolved in *Liphistius* and *Latrodectus*, or that ECPs were present in the common ancestor of all extant spiders. Given the striking similarity of amino acids over a long region (∼200 residues) and lack of significant similarity to any other proteins in the NCBI nr database, it seems unlikely that ECPs evolved convergently in mesotheles and in theridiid araneomorphs (Figure 2). Thus, we propose homology of *Latrodectus* ECPs and *Liphistius* ECPLs. However, a recent study on silk gland transcriptomes from the mygalomorph, *Actinopus sp*., and an orbicularian araneomorph, *Gasteracantha cancriformis*, also did not report ECPs [40]. If our hypothesis of homology is correct, ECPs must have been lost independently in many spider lineages. Alternatively, ECPs may be highly restricted in their timing of expression, eluding detection in most cDNA libraries. With the completion of spider genome sequences in the future, it will be possible to discern the presence, absence, or pseudogenization of ECPL genes in various spider taxa, and test the hypothesis of homology between the distantly related ECP and ECPL genes. In particular, synteny could provide additional evidence for orthology of ECPs from *Latrodectus* and ECPLs from *Liphistius*.

Both *Latrodectus* ECPs and *Liphistius* ECPLs are cysteine rich, with many cysteine positions conserved within and across species (Figure 2; [30], [31]). However, *Liphistius* ECPLs are significantly shorter than *Latrodectus* ECPs, lacking most of the extensive repetitive region seen in *Latrodectus* ECPs. While the timing and specificity of ECPL expression in *Liphistius* is uncertain, the physiochemical conservation of 73% of amino acids at sites that are present in at least one ECPL and ECP suggests that these ECPLs have a cross-linking role in silk fiber formation similar to that proposed for ECPs [31].

While mesotheles have high ECPL diversity, our cDNA screen suggests a low spidroin diversity, as only a single spidroin type (fib1) was detected in our *Liphistius* cDNA library. The presence of a spidroin in a mesothele confirms that the spidroin gene family evolved very early in Araneae and has an important role in silk production for all major spider groups that have been studied to date. Whether the *Liphistius* spidroin forms complexes with the ECPLs is currently unknown. In *Latrodectus*, ECPs form trimeric complexes with the N-terminal region of tubuliform spidroins (TuSp1) to make the outer silk wrapped around eggs [31]. The N-terminal region of *Liphistius* fib1 has not been characterized, but there are three cysteines in the C-terminal region that may allow for disulfide bonds with the ECPLs, as well as between fib1 monomers. Phylogenetic analyses did not recover a close relationship between *Liphistius* fib1 and TuSp1, indicating that TuSp1 is the result of spidroin duplication after the split of Opisthothelae from Mesothelae (Figures 1, 3). This implies that ECPs evolved prior to TuSp1. Thus, ECPs likely first were incorporated into silk fibers made with spidroins that were serving a more general purpose, and later became incorporated into *Latrodectus* tubuliform silk fibers, which are specialized for egg case construction.

### Spidroin Evolution

The most parsimonious rooting of the spidroin gene tree using reconciliation analysis indicates that *Liphistius* fib1 is sister to all other spidroins (Figure 3). Alternative less parsimonious rootings of the spidroin gene tree are consistent with spidroin gene family duplications occurring prior to the split of mesotheles and opisthotheles (Table S2). While mesotheles may have retained a single spidroin type, opisthotheles underwent an extensive diversification of spidroins very early in their history. Non-monophyly of araneomorph spidroins and of mygalomorph spidroins confirms that duplications occurred prior to the initial split of opisthotheles [21], [40]. The common ancestor of opisthotheles minimally had five spidroin paralogs (Figure 3). These five paralogous gene lineages are now represented by 1) *Hypochilus* fib1, 2) a clade consisting of two mygalomorph spidroins and four haplogyne spidroins, 3) a clade consisting of orbicularian aciniform spidroins plus *Hypochilus* fib2 and orbicularian tubuliform spidroins plus *Plectreurys* fib3, 4) a clade consisting of the remaining mygalomorph spidroins and orbicularian pyriform spidroins plus *Plectreurys* fib4, and 5) a clade consisting of major and minor ampullates, orbicularian flagelliforms, and three additional *Deinopis* spidroins (Figure 3).

The spidroin gene tree allows for inference of the duplication history of spidroins and how the origins of these different gene copies relate to the diversification of silk glands and to the evolution of spigot morphology. Mygalomorphs generally have a single spigot type and silk glands that are largely uniform and acinous in shape, which is thought to be the ancestral condition for spiders [34], [35], [38]. Given the diversity of spidroins hypothesized in the opisthothele common ancestor, spidroin diversification preceded the evolution of morphologically distinct silk glands (Figure 3).

The last common ancestor of araneomorphs is believed to have possessed ampullate, aciniform, pyriform, and cribellate silk glands and differentiated spigot types for each of these glands [2], [32], [54]. Spidroin ortholog groups associated with these glands are represented in the opisthothele common ancestor, with the exception of the cribellate spidroins, which to date have not been identified (Figure 3). Additionally, tubuliform and aciniform spidroins are inferred to have resulted from gene duplication before the diversification of Araneomorphae. Tubuliform spigots are a synapomorphy for entelygyne araneomorphs, yet both tubuliform and aciniform spidroins have non-entelegyne relatives, consistent with spidroin diversification preceding the evolution of the morphologically distinct tubuliform gland and spigot type [54]–[56]. Based on our gene tree, the flagelliform, major ampullate, and minor ampullate spidroins appear to have diversified within Entelegynae. For the cDNA libraries from non-entelgyne spiders screened in this study, fibroins closely related to ampullate and flagelliform fibroins were not found.

As in two recent studies, we did not recover monophyly of mygalomorph spidroins in our phylogenetic analyses [21], [40]. In contrast to these other studies, our increased taxonomic sampling reveals that both of the mygalomorph spidroin clades include atypoid and non-atypoid spidroins, indicating that ancient spidroin duplicates may be retained in different mygalomorph taxa, as seen in *Aphonopelma* (Figure 3). However, some mygalomorph taxa, such as *Bothriocyrtum*, retain spidroin copies that are very similar to each other, consistent with recent gene duplication or homogenization via concerted evolution in this mygalomorph lineage.

Mesothele and mygalomorph species have evolved a wide variety of web architectures, including sheet-webs, purse-webs, and trapdoors [3]. Assuming that the spidroins we have characterized from these taxa are those used to construct their webs, the relationship between different web shapes and the spidroins used to construct them appears to be highly variable. In many cases, closely related spidroin proteins may be used in the construction of very different web architectures. For example, *Aliatypus* spiders construct trapdoors, yet their spidroin is most closely related to the spidroins of *Hexura* and *Megahexura*, which construct sheet-webs (Figures 3, S1). On the other hand, very similar architectures may be built from very divergent spidroins. *Liphistius*, *Aliatypus*, *Aptostichus*, and *Bothriocyrtum* have convergently evolved trapdoors, and the spidroins found from most of these spiders are not closely related. Thus, the ability of mesothele and mygalomorph species to produce different web architectures does not seem to be constrained by the silk proteins produced.

### Evolution of Spidroin Repeat Regions

Our analyses reveal very low nucleotide sequence variability among repeat units within a particular spidroin gene. Even *Hypochilus* fib2 and *Plectreurys* fib3, which are the only reported spidroins composed of two different ensemble repeat types (a tandem array of a particular ensemble repeat followed by a tandem array of a different ensemble repeat), have high sequence similarity across ensemble repeats of the same type. Homogenization of repeats is consistent with concerted evolution via intragenic gene conversion or unequal crossing over, and is a pattern typical of spidroins reported from mygalomorph and araneomorph spiders [18]–[21], [26], [57]. The homogenization of repeats seen in *Liphistius* (Figure 6) indicates that a gene architecture of tandemly arranged, homogenized repeats is an ancestral feature for spidroins.

*Liphistius* fib1 and nearly all mygalomorph spidroin repeats described here are ∼180 AA long (157–194 AA; Figure 4). The exceptions are *Antrodiaetus* fib1 (repeat length of 50 AA) and *Megahexura* fib1 (365 AA). The *Megahexura* fib1 repeat could have arisen from a doubling of the unit of homogenization (∼180 to ∼360 AA), which has been postulated for the large size of the *Euagrus* fib1 repeat (342 AA). The *Euagrus* fib1 repeat can be divided into two subrepeats of approximately equal size that are 56% identical [21]. This suggests that the *Euagrus* fib1 repeat arose from a change of the unit of homogenization from ∼170 to 342 AA. The *Megahexura* fib1 repeat (365 AA) cannot be divided into two subrepeats, suggesting that extensive sequence divergence has occurred between its putative ∼180 AA ancestral subrepeats. Further studies are needed to determine whether ∼180 AA is an optimal length for mygalomorph and mesothele silk production. At present, studies on recombinant silk production have focused on number of repeats and fiber formation, but not the influence of repeat size on fiber formation and mechanical properties [58], [59].

Alanine, glycine, and serine are three of the major amino acid components of spider silks and the silks of other arthropods [29], [60]; for the spidroins analyzed here, these three amino acids account for, on average, 64% of the total amino acid content of the repetitive region. The percentages of these common amino acids vary considerably across the spidroin gene tree (Figure 5, Table S2). For most spidroins, alanine levels fall within the range of 20–35%. This is also exhibited by the *Liphistius* spidroin (26.5% alanine), and ancestral state reconstruction posits 26–36% as the primitive condition for spider silks. The best fitting model for alanine, under the most conservative asymmetry threshold in CoMET, indicates that the branching patterns in the spidroin C-terminal tree and DNA sequence divergence level between C-terminal encoding regions predicts the divergence level of alanine percentage in the repetitive regions [52].

The heat map of the percent compositions of serine and glycine across the spidroin gene tree indicates that they contrast strikingly with each other (Figure 5). *Liphistius* fib1, most myaglomorph spidroins, and most non-ampullate and non-flagelliform araneomorph spidroins exhibit moderately high serine levels, but are deficient in glycine. In contrast, ampullate and flagelliform spidroins show high levels of glycine and low levels of serine. The best fitting CoMET model determined for glycine percentage suggests that at branching events in the spidroin gene tree, one spidroin retains the ancestral glycine level while the other descendant gene lineage diverges [52]. Punctuated evolution of glycine could be due to selection for sequence encoding glycine rich motifs, spread rapidly throughout the gene by concerted evolution, and maintained thereafter by stabilizing selection. Glycine rich motifs are known to contribute to the high tensile strength and extensibility of major ampullate and flagelliform silk fibers, respectively [29]. As was the case for alanine, the best fitting model selected for serine percentage indicates that change in serine composition more closely reflects the spidroin relationships and level of spidroin C-terminal sequence divergences [52]. Therefore, the CoMET models suggest that, given the spidroin tree, alanine and serine percentages change gradually, whereas glycine levels exhibit a pattern of large change followed by stasis.

Spider silks vary greatly in mechanical performance across species and among silks associated with different gland types [14], [61], [62]. Tensile testing of silks from representatives of *Liphistius* and mygalomorphs has shown that these silks have lower tensile strength than major ampullate silks and lack the high extensibility of flagelliform silks [14], [63]. Thus far, silk mechanical properties have only been tested on a few mesothele species and theraphosid mygalomorphs (tarantulas). Our study reveals mygalomorph silk proteins with distinct molecular architectures that may enable unique, and perhaps exceptional, mechanical properties. For example, *Antrodiaetus* expresses two silk encoding genes, one of which (fib1) encodes a protein with a glycine percentage of ∼30%, which is more comparable to major ampullate and minor ampullate silks (∼24–45%) than theraphosid silks (<10%). Also, the repeat length encoded by *Megahexura* fib1 is ∼365 AA, which is well above the known range of repeat lengths encoded by theraphosid spidroin genes (157–186 AA). Thus, broader examination of silk mechanical properties in different mygalomorphs is warranted.

Mesotheles and mygalomorphs mostly use their silks to line their burrows, construct retreats, make egg sacs, and extend their sensory area. Exceptionally extensible or strong silk may not be advantageous for these purposes [3]. These spiders rely on their size, power, and robust fangs to capture ground dwelling prey, and there is little need for silks capable of absorbing kinetic energy from flying insects. Instead, selection in mesothele and mygalomorph lineages may favor durable silks that are optimized for stability in subterranean conditions or for sensitivity in detection of vibrations from prey. The new silk genes we have found can be used to further investigate silk mechanical and functional properties and how these relate to the subterranean lifestyle of mesotheles and mygalomorphs.

### Conclusion

Analysis of silk gland expression libraries from mesothele, paleocribellate, and mygalomorph spiders greatly clarifies the evolutionary history of silk in Araneae. The discovery of mesothele ECPL sequences that share conserved regions with *Latrodectus* ECPs suggests that these loci comprise a gene family which has been associated with silk production in spiders for >380 million years. Further research is needed to determine the phylogenetic breadth of this gene family in spiders, as well as how ECPs functionally interact with members of the spidroin gene family. Phylogenetic analysis of our new data from Mesothelae, Mygalomorphae, and Paleocribelletae suggests that the most recent common ancestor of all extant spiders had a single spidroin, and that diversification of spidroins by gene duplication had already occurred prior to the divergence of mygalomorphs and araneomorphs. We also found that repeat regions vary considerably in amino acid composition across different spidroin types. The punctuated pattern of change in glycine percentage could be due to selection for improved mechanical properties enabled by these characteristics, facilitated by concerted evolution quickly spreading desirable protein coding motifs throughout a spidroin gene.

Mesotheles and mygalomorphs construct a wide variety of web shapes and burrow entrance architectures. Considering the ecological function of mygalomorph and mesothele silks, selection on silk from these spiders may have favored properties associated with the largely subterranean niche they fill, such as durability for burrow maintenance and vibration transmission for prey capture [3]. The diversity of silk genes we have uncovered in mesotheles and mygalomorphs highlights the need for further exploration into the phylogenetic diversity of spiders for silk genes that encode unique silk mechanical properties.

## Materials and Methods

### Taxonomic Sampling

Our taxonomic sampling was aimed at covering phylogenetic diversity and surveying a variety of web architectures. The mesothele representative, *Liphistius malayanus*, constructs a subterranean burrow with a trapdoor and radiating sensory lines. Six species of mygalomorphs were sampled. From the Atypoidea clade, which is the sister group to remaining mygalomorphs [9], [48], the following species were selected, with web constructs in parentheses: *Megahexura fulva* (sheet-web), *Hexura picea* (sheet-web), *Sphodros rufipes* (purse-web), and *Antrodiaetus riversi* (burrow with turret-like entrance). We sampled two non-atypoid mygalomorphs from the family Theraphosidae, *Aphonopelma seemanni*, a ground dweller (burrow/sheet-web), and *Poecilotheria regalis*, an arboreal species (sheet-web). Finally, we included the lamp-shade web spider, *Hypochilus thorelli*, which is a member of the basal araneomorph lineage, Paleocribellatae.

All specimens used in our study were obtained from pet stores or were collected on public, unprotected lands. Additionally, no species used in this study is protected or endangered. Thus, no specific permits were required for the described field studies.

### cDNA Library Construction and Screening

We followed the cDNA library construction methods described in Garb et al. [21]. Briefly, each spider was anesthetized with CO_2_ and then the entire set of silk glands was removed intact. The silk glands were frozen in liquid nitrogen and stored at −80°C. With the exception of the two theraphosids, glands from multiple individuals of the same species were combined to obtain sufficient tissue. Total RNA was extracted using TRIzol (Invitrogen, Carlsbad, CA) and the RNeasy Minikit (Qiagen, Valencia, CA). We isolated mRNA from total RNA using Dynal magnetic beads with oligo-(dT) anchors (Invitrogen). Double-stranded cDNA was constructed using the Superscript Choice protocol (Invitrogen), and then size selected for large fragments using Chroma Spin 1000 columns (Clontech, Mountain View, CA). The size-selected cDNA was ligated into pZErO 2.0 vectors that had been digested with EcoRV, and then transformed into TOP10 *Escherichia coli* (Invitrogen). For each species, we arrayed ∼1400–1700 cDNA clones into 96-well microtiter plates. The libraries were stored at −80°C.

We screened approximately one third of each library using the method of Beuken, Vink, and Bruggeman [64] and sequenced clones containing inserts ≥500 base pairs with T7 and Sp6 universal primers. Sequences were compared to the NCBI nr database using BLASTX [46] to identify potential silk homologs. Libraries were also replicated onto nylon filters and probed with γ^32^ P-labeled oligonucleotides. All libraries were screened with GCDGCDGCDGCDGCDGC and CCWGCWCCWGCWCCWGCWCC, which were designed based on motifs common to spidroins [18], [20], [65]. Additionally, libraries were screened with taxon specific probes designed from the end sequences of the size-selected clones. For putative *Liphistius* Egg Case Proteins (ECPs), the following probes were developed: 1) TAGTAATAAGTTCCATCGCA, 2) GCAAGGATTATAAGGATG, 3) CTTACCCTCTCCACATTCAGT, 4) GGTTTAACTTTGTTGGCGTC, 5) GGGGTCGTAAAATGATTGATA, 6) ACATTGGTTCTTTTTGTAGCA, and 7) GTTCTTGTCGTAGCATTTGTA. Probes designed from putative spidroins were 8) AAAAGCAGTGGCAGTGGCTTC, 9) CCCCTAAAATAGGTATTCTGATA (8, 9 for *Liphistius*); 10) GCCGTATGATGCTGACTGTAG, 11) TGCTGATGCGGCGGCTTG, 12) GCTTGCATAGGCTGAGGC (10–12 for *Megahexura*); 13) TATATCAGTTCCATATGGTCC, 14) GGATCGAAAACGTTGTGAAA, 15) AGCTGCTTCATTTGCTGTGTT, 16) CTTACCACAGGCGTAACC (13–16 for *Hexura*); 17) GCCGCTGCATCGGCGTAGGC, 18) AATGCAAATGCGATGGCATA, 19) CAACACACCACTCAATCCAGA (17–19 for *Sphodros*); 20) GCTCCTTCWCTMCCATATCCTCC, 21) GCTTCAGCATAYGCTTTTGC, 22) TCTRGCATAACTAGCGGCATC, 23) GTAAACTGATTCGAATTCGTC (20–23 for *Antrodiaetus*); 24) TTATCACACATCATTTTTCC (24 for *Aphonopelma*); 25) CATGGCAGAGGGTATCAGGT, 26) AGTGTAATTTGCAATGCC, 27) GCAAGAGCAATGGCGTTTCC, 28) ATAGGCATAAGCACCAGCGTT, 29) GTAAGCATAAGCCTCGGCTCC (25–29 for *Poecilotheria*); 30) AGCTCCWGCACTTGCNCCACT (30 for *Hypochilus*).

All positive clones were sequenced using T7 and Sp6 universal primers. Based on these sequences, clones that had the same translated carboxyl (C) terminal region were grouped with each other. For each group, the clone with the longest insert was selected for complete characterization. Because the inserts contained repetitive nucleotide sequence, a primer walking approach was not feasible. Instead, each selected clone was bidirectionally sequenced in its entirety using the transposon-based GPS-1 Genome Priming System (NEB, Ipswich, MA) or EZ-Tn5 Kit (Epicentre, Madison, WI).

### Alignment of Egg Case Proteins

Putative *Liphistius malayanus* ECPs were aligned with *Latrodectus hesperus* ECP-1 (AY994149) and ECP-2 (DQ341220) using MUSCLE with default settings [66]. The alignment was imported into GeneDoc 2.7.0 [67] and physiochemically conserved sites were highlighted.

### Phylogenetic Analyses

Phylogenetic analyses were conducted on a dataset of C-terminal encoding regions from published spidroins and those reported here. Spidroins from GenBank were selected to represent different silk glands and phylogenetic diversity. From Araneomorphae, we included *Argiope trifasciata AcSp1* (accession number AY426339), *Flag* (AF350264), and *pyriform* (GQ980328; referred to as *PySp1* in this paper); *Deinopis spinosa Flag* (DQ399325), *fibroin 1a* (DQ399326), *fibroin 1b* (DQ399327), *fibroin 2* (DQ399323), *MaSp2a* (DQ399328), *MaSp2b* (DQ399329), *MiSp1* (DQ399324), and *TuSp1* (AY953073); *Diguetia canities MaSp-like* (HM752567) and MaSp (HM752565; referred to as *MaSp-like2* in this paper); *Dolomedes tenebrosus Dtfib1* (AF350269) and *Dtfib2* (AF350270); *Latrodectus hesperus AcSp1* (EU025854), *MaSp1* (DQ409057), *MaSp2* (EF595245), *PySp1* (FJ973621), and *TuSp1* (AY953070); *Nephila clavipes Flag* (AF027973), *MaSp1* (AY654292), *MaSp2* (M92913), *MiSp1* (AF027735), *pyriform* (GQ980330; referred to here as *PySp1*), and *TuSp1* (AY855102); *Peucetia viridans MaSp1* (GU306168); *Plectreurys tristis fibroin 1* (AF350281), *fibroin 2* (AF350282), *fibroin 3* (AF350283), and *fibroin 4* (AF350284); and *Uloborus diversus AcSp1* (DQ399333), *MaSp1* (DQ399331), *MaSp2* (DQ399334), *MiSp* (DQ399332), and *TuSp1* (AY953072). From Mygalomorphae, we included *Aliatypus gulosus fibroin 1* (EU117159); *Aptostichus* sp. *fibroin 1* (EU117160) and *fibroin 2* (EU117161); *Avicularia juruensis spidroin 1a* (EU652181; referred to as *fib1a* in this study), *1b* (EU652182; referred to as *fib1b* in this study), and *1c* (EU652183; referred to as *fib1c* in this study); *Bothriocyrtum californicum fibroin 1* (EU117162), *fibroin 2* (EU117163), and *fibroin 3* (EU117164); and *Euagrus chisoseus fibroin 1* (AF350271). The C-terminal regions were aligned using MUSCLE under default parameters [66] followed by manual adjustment. C-terminal encoding DNA sequences were aligned according to the amino acid alignment with PAL2NAL [68].

We did not include *Avicularia juruensis spidroin 2* (EU652184) in our final analyses, as it is potentially an experimental artifact. A BLASTN search of the *Avicularia spidroin 2* C-terminal region resulted in only two hits. These hits (accessions AF350267, AY365020) were *MaSp2* sequences from two species of the orbicularian, *Argiope,* and had extremely small, highly significant E values (<1e-63). Phylogenetic analysis grouped this sequence with araneoid major ampullate sequences [39]. This result could not be corroborated as close relatives of *Avicularia spidroin 2* were not found in any of 10 mygalomorph cDNA libraries ([18], [21] this study); nor did Prosdocimi et al. [40] recover major ampullate-like spidroins from the silk gland transcriptome of the mygalomorph, *Actinopus sp*.

We conducted phylogenetic analyses using ML and Bayesian methods. Analyses were conducted on DNA data with gaps coded using the ‘Simple’ method following Simmons and Ochoterena [69]. Analyses were conducted through the CIPRES web server [70]. Likelihood searches for the best tree and bootstrap were performed simultaneously with 1000 replicates using RAxML v. 7.2.8 [71]–[73]. Analyses were performed with the data partitioned by codon position, using the GTR+γ model for each partition, following RAxML program author recommendations. Coded gaps were treated as binary data and as a separate data partition.

Bayesian analyses were conducted using MrBayes v. 3.1.2 [74], [75]. DNA substitution models were determined for each codon position (position 1: HKY+I+γ, position 2: GTR+I+ γ, position 3: GTR+ γ) using MrModeltest v. 2.3 [76]. The restriction site (binary) model with variable ascertainment bias was used for the coded gap characters [77]. Two simultaneous searches were run for at least 10 million generations, with trees and parameters sampled from four MCMC chains every 1000^th^ generation. Partitions (codon positions and binary characters) were unlinked and substitution rates of evolution among partitions were allowed to vary. Analyses were considered complete when the standard deviation of split frequencies between the two searches was below 0.01 [77]. The first forty percent of samples were treated as burnin and discarded. Bayesian posterior probabilities (PP) were used to assess clade support.

Likelihood and Bayesian analyses were also conducted with constraints placed for each gland-associated spidroin group (i.e., minor ampullate, major ampullate, flagelliform, tubuliform, pyriform, and aciniform gland types; Figure S1; Table S1). Our higher-level sampling was not intended to establish monophyly of each of the gland associated spidroin groups; rather we aimed to determine the phylogenetic placements of the gland associated spidroin groups among spidroins from across the spider phylogeny. For minor ampullate, flagelliform, tubuliform, pyriform, and aciniform glands, spidroins have been reported from only a few species, while major ampullate spidroins are more widely known. Our sample of major ampullate spidroins is not comprehensive because we focused on sampling species for which multiple spidroins had been characterized. Using N and C-terminal sequences, Garb et al. [23] recovered monophyletic groups for each of tubuliforms, flagelliforms, and minor ampullates in parsimony and Bayesian analyses. Entelegyne major ampullates spidroins were also recovered as monophyletic in their Bayesian analysis. N-terminal sequences have not been reported for aciniform and pyriform gland associated spidroins, or from any mygalomorph spidroins except for one (*Bothriocyrtum californicum* fib1). We did not recover N-terminal sequences in any of our libraries; thus we did not include published N-terminal sequences in our analyses. An SH test [47] using RAxML with the log likelihood values from the ML analyses was preformed to compare the constrained and unconstrained tree topologies.

The constrained ML spidroin gene tree was reconciled with a species tree based on hypothesized phylogenetic relationships [9], [48], [78] using the program GeneTree 1.3 [79]. Spidroins lack a non-spider outgroup. Thus, rooting of the spidroin gene tree was based on the minimization of total gene duplications plus losses.

### Characterization of Spidroin Non-Terminal Regions

Tandem repeats in spidroin protein sequences were identified using XSTREAM under default settings [49] and by eye. Consensus repeat sequences and their lengths for each spidroin were determined based on 50% majority rule with ambiguities indicated by an X (Figure 4). We also determined the amino acid compositions of spidroin repetitive regions with MacVector 7.2 (Accelrys Inc., San Diego, CA; Table S2).

Using the ML C-terminal tree from the analysis with gland associated spidroins constrained, we performed continuous character, ancestral state reconstructions for amino acid compositions. Reconstructions were done using parsimony under the linear cost assumption in Mesquite v. 2.74 [80]. Additionally, the Mesquite module, CoMET, was used to calculate the likelihood of observing the continuous data given the entire C-terminal tree (all branching events and branch lengths) under nine different models of evolution [51]. These models include pure phylogenetic, non-phylogenetic, or punctuated average, in combination with distance, equal, or free [52]. The best fitting model was determined by the Akaike Information Criterion [53]. CoMET analyses were run with thresholds of 100 and 1000 for comparison of the pure phylogenetic and punctuated average models. The punctuated average model was favored if the data was indicated to have evolved from branching events where the branch lengths were 100 or, more conservatively, 1000 times longer than their corresponding sister branch lengths (CoMET User’s Guide, Feb. 2006).

## Supporting Information

Figure S1 **Spidroin gene tree based on ML analysis of the carboxy-terminal encoding region**. In the analysis, gaps were coded as binary characters and monophyly of some groups was constrained (see Methods). Numbers at nodes correspond to information in Tables S1 and S2. Node numbers indicated in red are constrained nodes. Green dots indicate nodes that do not conflict between the analysis with node constraints and the unconstrained ML analysis. Dots at terminal nodes indicate web type constructed by the taxa from which the spidroin sequence was obtained (red = trapdoor, blue = sheetweb, purple = purseweb, teal = turret). Hash marks on branch indicate arbitrary shortening of branch for figure quality purposes. Brackets indicate taxonomic group of spiders from which spidroins were characterized and select spidroin clades using the following abbreviations: Me = Mesothelae, My = Mygalomorphae, Ar = Araneomorphae, AcSp = Aciniform, TuSp = Tubuliform, PySp = Pyriform, MaSp = Major ampullate, MiSp = Minor ampullate, Flag = Flagelliform.
(TIF)

Table S1 **Node support (ML bootstrap percentage (BP) and Bayesian posterior probability (PP)) for phylogenetic analyses**. Node numbers refer to the phylogeny in Figures 3 and S1. Dashes refer to nodes with <50 BS or 0.5 PP support.
(PDF)

Table S2 **Continuous character data and alternative reconciliation based outgroups for ML constrained tree**. Ancestral state parsimony optimization was determined by Mesquite v. 2.74 [80]. Node numbers refer to the phylogeny in Figures 3, 5, and S1.
(PDF)
