# Supplementary figures and images for: Early Events in the Evolution of Spider Silk Genes
Source: PLoS One. 2012 Jun 22;7(6):e38084. doi: 10.1371/journal.pone.0038084 (PMC3382249; doi:10.1371/journal.pone.0038084)

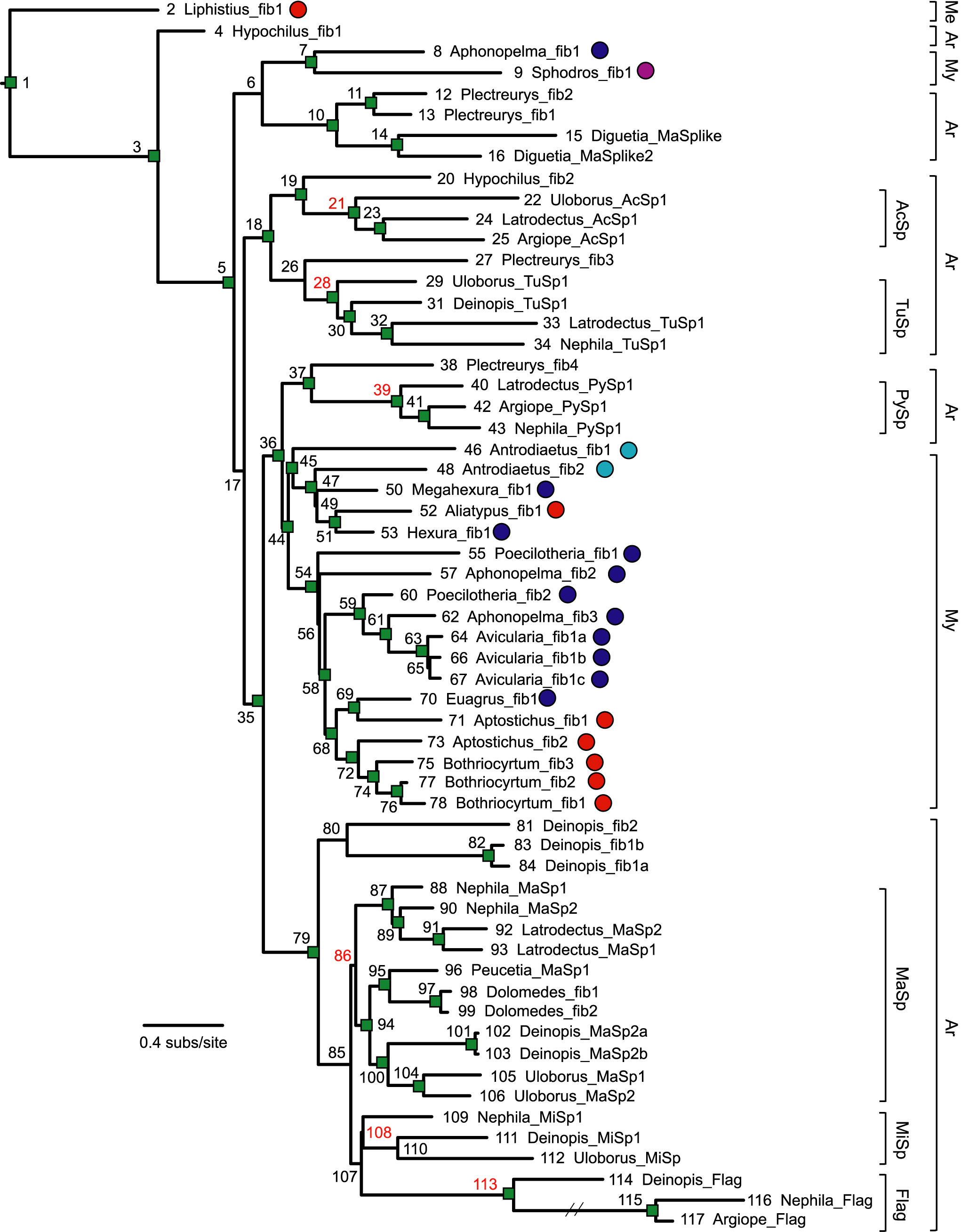

Supplement: Figure S1 — Spidroin gene tree based on ML analysis of the carboxy-terminal encoding region. In the analysis, gaps were coded as binary characters and monophyly of some groups was constrained (see Methods). Numbers at nodes correspond to information in Tables S1 and S2. Node numbers indicated in red are constrained nodes. Green dots indicate nodes that do not conflict between the analysis with node constraints and the unconstrained ML analysis. Dots at terminal nodes indicate web type constructed by the taxa from which the spidroin sequence was obtained (red = trapdoor, blue = sheetweb, purple = purseweb, teal = turret). Hash marks on branch indicate arbitrary shortening of branch for figure quality purposes. Brackets indicate taxonomic group of spiders from which spidroins were characterized and select spidroin clades using the following abbreviations: Me = Mesothelae, My = Mygalomorphae, Ar = Araneomorphae, AcSp = Aciniform, TuSp = Tubuliform, PySp = Pyriform, MaSp = Major ampullate, MiSp = Minor ampullate, Flag = Flagelliform. (TIF) [file pone.0038084.s001.tif]
