# Supplementary material for: Early Events in the Evolution of Spider Silk Genes
Source: PLoS One. 2012 Jun 22;7(6):e38084. doi: 10.1371/journal.pone.0038084 (PMC3382249; doi:10.1371/journal.pone.0038084)
Supplement: Table S1 — Node support (ML bootstrap percentage (BP) and Bayesian posterior probability (PP)) for phylogenetic analyses. Node numbers refer to the phylogeny in Figures 3 and S1. Dashes refer to nodes with <50 BS or 0.5 PP support. (PDF) [file pone.0038084.s002.pdf]

## Supplementary Table

Table S1. **Node support (ML bootstrap percentage (BP) and Bayesian posterior probability (PP)) for phylogenetic analyses**. Node numbers refer to the phylogeny in Figures 3 and S1. Dashes refer to nodes with <50 BS or 0.5 PP support.

| Node# | ML | | | Bayes | |
|---|---|---|---|---|---|
| | | | | | |
| | Constrained (C) | Unconstrained | | Constrained (C) | Unconstrained |
| 1 | - | - | | - | - |
| 3 | - | - | | - | - |
| 5 | - | - | | 0.78 | 0.8 |
| 6 | - | - | | - | - |
| 7 | 65 | 67 | | 0.97 | 0.98 |
| 10 | 83 | 79 | | 1.0 | 0.99 |
| 11 | 92 | 89 | | 1.0 | 1.0 |
| 14 | 87 | 87 | | 1.0 | 1.0 |
| 17 | - | - | | - | - |
| 18 | - | - | | - | - |
| 19 | - | - | | 0.57 | 0.66 |
| 21 | C, 97 | 90 | | C, 1.0 | 1.0 |
| 23 | - | 53 | | - | 0.56 |
| 26 | - | - | | 0.54 | 0.5 |
| 28 | C, 74 | - | | C, 1.0 | 0.97 |
| 30 | - | - | | 0.51 | 0.53 |
| 32 | 68 | 56 | | 0.94 | 0.93 |
| 35 | - | - | | 0.74 | 0.77 |
| 36 | - | - | | - | - |
| 37 | - | - | | 0.59 | 0.62 |
| 39 | C, 94 | 92 | | C, 1.0 | 1.0 |
| 41 | 75 | 75 | | 0.94 | 0.95 |
| 44 | - | - | | - | - |
| 45 | - | - | | - | - |
| 47 | - | - | | 0.65 | 0.59 |
| 49 | - | - | | - | - |
| 51 | 55 | 52 | | 0.91 | 0.9 |
| 54 | - | - | | 0.67 | 0.67 |
| 56 | - | - | | - | 0.5 |
| 58 | - | - | | - | - |
| 59 | 82 | 82 | | 1.0 | 1.0 |
| 61 | 83 | 80 | | 1.0 | 1.0 |
| 63 | 93 | 93 | | 1.0 | 1.0 |
| 65 | 56 | - | | - | - |
| 68 | - | - | | - | - |
| 69 | 70 | 72 | | 0.93 | 0.93 |

| | | | | | |
|---|---|---|---|---|---|
| 72 | - | - | | 0.94 | 0.95 |
| 74 | 59 | 54 | | 0.97 | 0.97 |
| 76 | 98 | 97 | | 1.0 | 1.0 |
| 79 | - | - | | 0.98 | 0.98 |
| 80 | - | - | | 0.84 | 0.61 |
| 82 | 100 | 100 | | 1.0 | 1.0 |
| 85 | - | - | | 0.61 | 0.5 |
| 86 | C, - | - | | C, 1.0 | - |
| 87 | 82 | 69 | | 1.0 | 0.99 |
| 89 | 67 | 61 | | 0.81 | 0.82 |
| 91 | 98 | 98 | | 1.0 | 1.0 |
| 94 | - | - | | 0.94 | 0.71 |
| 95 | 63 | - | | 0.99 | 0.94 |
| 97 | 95 | 94 | | 1.0 | 1.0 |
| 100 | 82 | 77 | | 0.96 | 0.96 |
| 101 | 100 | 100 | | 1.0 | 1.0 |
| 104 | 99 | 99 | | 1.0 | 1.0 |
| 107 | - | - | | - | - |
| 108 | C, 55 | - | | C, 1.0 | - |
| 110 | - | - | | 0.69 | - |
| 113 | C, 91 | - | | C, 1.0 | 0.99 |
| 115 | 100 | 100 | | 1.0 | 1.0 |
