# Supplementary material for: Early Events in the Evolution of Spider Silk Genes
Source: PLoS One. 2012 Jun 22;7(6):e38084. doi: 10.1371/journal.pone.0038084 (PMC3382249; doi:10.1371/journal.pone.0038084)
Supplement: Table S2 — Continuous character data and alternative reconciliation based outgroups for ML constrained tree. Ancestral state parsimony optimization was determined by Mesquite v. 2.74 [80]. Node numbers refer to the phylogeny in Figures 3, 5, and S1. (PDF) [file pone.0038084.s003.pdf]

Supplementary Table

Table S2. **Continuous character data and alternative reconciliation based outgroups for ML constrained tree**. Ancestral state parsimony optimization was determined by Mesquite v. 2.74 [80]. Node numbers refer to the phylogeny in Figures 3, 5, and S1.

Node#	Alanine %	Glycine %	Serine %	Alternative Rooting/ Outgroup
				duplications/ losses/ deep coalescence events
1	26.5-36.44	3.15-12.83	17.18-19.03	
2 (*Liphistius*_fib1)	26.5	3.15	19.03	31/69/81
3	26.5-36.44	9.69-12.83	17.18-18.94	
4 (*Hypochilus*_fib1)	40.28	23.7	9.72	31/70/82
5	26.5-36.44	9.69-12.83	17.18-18.94	31/70/82
6	26.5-36.44	9.69-12.83	17.18-18.94	31/71/83
7	26.5-36.44	7.13	18.94	
8 (*Aphonopelma*_fib1)	8.15	7.13	18.94	
9 (*Sphodros*_fib1)	39.07	4.52	24.78	
10	29.1-36.44	13.15-19.0	12.64-18.94	
11	29.1-36.44	13.15-19.0	12.64-18.94	
12 (*Plectreurys*_fib2)	29.1	13.15	20.09	
13 (*Plectreurys*_fib1)	41.19	27.1	12.64	
14	36.44	19	11.74	
15 (*Diguetia*_MaSplike)	36.44	30.97	11.74	
16 (*Diguetia*_MaSplike2)	54.75	19	9.95	
17	25.26-29.65	9.69-12.83	17.18-18.94	31/71/83
18	25.26-29.65	9.69-12.83	20.53-21.46	31/72/84
19	25.26-29.65	9.69-12.83	20.53-21.46	31/74/86
20 (*Hypochilus*_fib2)	33.68	13.51	20.53	
21	14.67	9.69-12.83	20.53-21.46	
22 (*Uloborus*_AcSp1)	14.67	7.29	26.63	
23	14.15	12.83	20.53-21.46	
24 (*Latrodectus*_AcSp1)	13.43	12.83	13.63	
25 (*Argiope*_AcSp1)	14.15	15.5	21.46	
26	25.26-29.65	9.69	22.11-29.91	31/74/86
27 (*Plectreurys*_fib3)	25.26	2.14	30.94	
28	29.21-29.65	9.69	22.11-29.91	
29 (*Uloborus*_TuSp1)	29.65	9.69	29.91	
30	29.21-29.65	9.69	22.11-26.86	

31 (*Deinopis*_TuSp1)	34.5	11.78	20.34	
32	29.21	8.52	22.11-26.86	
33 (*Latrodectus*_TuSp1)	26.01	6.75	26.86	
34 (*Nephila*_TuSp1)	29.21	8.52	22.11	
35	24.23-27.59	9.69-12.83	17.18-18.94	31/72/84
36	22.44-27.59	8.25-12.83	17.18-18.94	31/73/85
37	22.44-27.59	8.25-12.83	17.18-18.94	
38 (*Plectreurys*_fib4)	22.44	13.85	17.18	
39	22.44-27.59	1.97-5.41	17.18-18.94	
40 (*Latrodectus*_PySp1)	45.15	0	7.26	
41	17.69	1.97-5.41	26.6	
42 (*Argiope*_PySp1)	17.69	5.41	27.76	
43 (*Nephila*_PySp1)	13.79	1.97	26.6	
44	22.44-27.59	8.25-12.83	17.18-21.38	
45	23.9-28.39	8.25-12.83	17.18-21.38	
46 (*Antrodiaetus*_fib1)	28.39	29.66	2.97	
47	23.9-28.39	6.23-10.53	22.09-23.07	
48 (*Antrodiaetus*_fib2)	23.9	4.92	31.45	
49	35.17	6.23-10.53	22.09-23.07	
50 (*Megahexura*_fib1)	38.07	10.53	22.09	
51	?	?	?	
52 (*Aliatypus*_fib1)	35.17	6.23	23.07	
53 (*Hexura*_fib1)	?	?	?	
54	20.7-27.59	8.25-8.9	19.37-21.38	
55 (*Poecilotheria*_fib1)	20.7	6.46	23.57	
56	20.7-27.59	8.25-8.9	19.37-21.38	
57 (*Aphonopelma*_fib2)	17.8	8.9	19.37	
58	20.7-33.23	8.25-8.9	21.38	
59	26.03-33.23	8.25-8.9	21.38	
60 (*Poecilotheria*_fib2)	33.23	10.15	21.38	
61	26.03-33.23	8.25	21.38	
62 (*Aphonopelma*_fib3)	26.03	8.25	20.32	
63	30.65-34.51	8.24-8.25	21.74-24.9	
64 (*Avicularia*_fib1a)	34.51	8.24	24.9	
65	30.65-34.51	8.24-8.25	21.74-24.9	
66 (*Avicularia*_fib1b)	30.65	9.57	21.74	
67 (*Avicularia*_fib1c)	36.95	5.99	25.2	
68	20.7-33.23	6.98-8.9	22.58-22.81	
69	20.7-33.23	6.98-8.9	22.81	
70 (*Euagrus*_fib1)	38.57	11.75	24.92	
71 (*Aptostichus*_fib1)	18.02	6.98	22.81	
72	20.7-33.23	6.07-6.19	22.58-22.81	
73 (*Aptostichus*_fib2)	19.97	6.07	23	
74	30.43-38.25	6.07-6.19	22.58-22.81	
75 (*Bothriocyrtum*_fib3)	38.25	6.19	21.04	
76	30.43-38.25	4.66	22.58-22.81	
77 (*Bothriocyrtum*_fib2)	38.28	4.66	22.58	
78 (*Bothriocyrtum*_fib1)	30.43	3.52	23.4	
79	24.23-27.59	40.22-40.91	6.9-18.94	31/73/85
80	24.23-27.59	40.91	6.9-18.94	31/77/89
81 (*Deinopis*_fib2)	16.48	40.91	19.13	31/84/96

82	27.59	46.06	6.9	31/84/96
83 (*Deinopis*_fib1b)	27.59	46.06	6.9	31/91/103
84 (*Deinopis*_fib1a)	31.14	48.23	4.86	31/91/103
85	24.23-27.59	40.22-40.91	6.7-6.73	31/77/89
86	24.23-27.59	40.22-40.91	6.7-6.73	31/81/93
87	24.23-32.25	40.22-40.91	6.12-6.73	31/85/97
88 (*Nephila*_MaSp1)	33.1	44.13	3.56	31/92/104
89	24.23-32.25	35.83-40.91	6.12-6.73	31/92/104
90 (*Nephila*_MaSp2)	22.26	35.09	7.55	
91	32.25	35.83-40.91	6.12	
92 (*Latrodectus*_MaSp2)	32.25	35.83	6.12	
93 (*Latrodectus*_MaSp1)	34.85	44.47	1.86	
94	24.23-26.5	40.22-40.91	6.7-6.73	31/85/97
95	24.23-26.5	43.55-43.6	6.7-6.73	
96 (*Peucetia*_MaSp1)	24.23	44.9	8.42	
97	24.23-26.5	43.55-43.6	6.7-6.73	
98 (*Dolomedes*_fib1)	23.12	43.55	6.51	
99 (*Dolomedes*_fib2)	28.96	43.6	6.73	
100	24.23-26.5	36.22-40.91	6.7-6.73	
101	23.61	36.22	4.46	
102 (*Deinopis*_MaSp2a)	19.41	36.22	4.45	
103 (*Deinopis*_MaSp2b)	23.61	32.97	4.46	
104	26.5	36.22-40.91	6.84	
105 (*Uloborus*_MaSp1)	26.92	44.23	8.65	
106 (*Uloborus*_MaSp2)	26.5	30.77	6.84	
107	24.23-27.59	40.22-40.91	6.7-6.73	31/81/93
108	24.23-35.47	40.22	6.7-6.73	31/86/98
109 (*Nephila*_MiSp1)	36.52	40.22	4.83	
110	24.23-35.47	34.16	10.73	
111 (*Deinopis*_MiSp1)	22.83	24.07	13.4	
112 (*Uloborus*_MiSp)	35.47	34.16	10.73	
113	5.49-12.01	45.58	6.7-6.73	31/86/98
114 (*Deinopis*_Flag)	3.49	45.58	6.7	
115	5.49-12.01	53	6.7-6.73	
116 (*Nephila*_Flag)	5.49	55.24	8.05	
117 (*Argiope*_Flag)	12.01	53	5.3	
